# Genetic diversity analysis and marker-trait associations in *Amaranthus* species

**DOI:** 10.1371/journal.pone.0267752

**Published:** 2022-05-12

**Authors:** Norain Jamalluddin, Festo J. Massawe, Sean Mayes, Wai Kuan Ho, Rachael C. Symonds

**Affiliations:** 1 Future Food Beacon, School of Biosciences, University of Nottingham Malaysia, Jalan Broga, Semenyih, Selangor, Malaysia; 2 Plant and Crop Sciences, Biosciences, University of Nottingham, Sutton Bonington Campus, Loughborough, Leicestershire, United Kingdom; 3 Crops for the Future (UK) CIC, NIAB, Cambridge, United Kingdom; 4 Liverpool John Moores University, School of Biological and Environmental Sciences, Liverpool, Merseyside, United Kingdom; Jeju National University, REPUBLIC OF KOREA

## Abstract

Amaranth (*Amaranthus* spp.) is a highly nutritious, underutilized vegetable and pseudo-cereal crop. It possesses diverse abiotic stress tolerance traits, is genetically diverse and highly phenotypically plastic, making it an ideal crop to thrive in a rapidly changing climate. Despite considerable genetic diversity there is a lack of detailed characterization of germplasm or population structures. The present study utilized the DArTSeq platform to determine the genetic relationships and population structure between 188 amaranth accessions from 18 agronomically important vegetable, grain, and weedy species. A total of 74, 303 SNP alleles were generated of which 63, 821 were physically mapped to the genome of the grain species *A*. *hypochondriacus*. Population structure was inferred in two steps. First, all 188 amaranth accessions comprised of 18 species and second, only 120 *A*. *tricolor* accessions. After SNP filtering, a total of 8,688 SNPs were generated on 181 amaranth accessions of 16 species and 9,789 SNPs generated on 118 *A*. *tricolor* accessions. Both SNP datasets produced three major sub-populations (K = 3) and generate consistent taxonomic classification of the amaranth sub-genera (*Amaranthus Amaranthus*, *Amaranthus Acnida* and *Amaranthus albersia*), although the accessions were poorly demarcated by geographical origin and morphological traits. *A*. *tricolor* accessions were well discriminated from other amaranth species. A genome-wide association study (GWAS) of 10 qualitative traits revealed an association between specific phenotypes and genetic variants within the genome and identified 22 marker trait associations (MTAs) and 100 MTAs (P≤0.01, P≤0.001) on 16 amaranth species and 118 *A*.*tricolor* datasets, respectively. The release of SNP markers from this panel has produced invaluable preliminary genetic information for phenotyping and cultivar improvement in amaranth species.

## Introduction

Climate predictions indicate that the agriculture sector in many parts of the world will be subjected to increasingly detrimental weather conditions such as droughts and elevated temperatures, directly impacting global food supply chains. A strategy to mitigate climate related agricultural losses is to diversify the food basket with a wide range of underutilized crop species with increased abiotic stress tolerance traits [1]. Amaranth (*Amaranthus* spp.), an ancient, nutrient-dense and climate-smart crop has high degree of genetic variation, environmental adaptability and phenotypic plasticity [2, 3]. Amaranth belongs to the Amaranthaceae family and is a C_4_ dicotyledonous plant [4]. It consists of approximately 60–70 species grouped into three sub-genera; *Amaranthus Albersia* (vegetable amaranth), *Amaranthus Amaranthus* (cultivated grain amaranth) and *Amaranthus Acnida* (weedy amaranth) [5].

*Amaranthus tricolor* is a leafy vegetable amaranth species, widely cultivated in South Asia and Africa [6, 7], and is an excellent source of vitamins, protein, carotenoid, minerals and antioxidants, greater than other leafy vegetables such as lettuce and spinach [2, 8]. *A*. *tricolor* has the capacity to alter its physiological characteristics in response to environmental changes, for instance, increasing transpiration efficiency [9] and accumulation of compatible solutes such as proline, in response to drought stress [10]. It also had high genetic and phenotypic diversity which may provide an excellent opportunity for varietal development with increased drought tolerance characteristics [11–13].

Correct genotypic identification and preservation of genetic variation in amaranth is important to maintain ecotypes with desired traits useful for breeding programmes. The assembly of very high-quality grain amaranth, *Amaranthus hypochondriacus* (“Plainsman” cultivar) sequence genome by [14] allows anchoring of genotyping-by-sequencing (GBS) markers for all the SNP loci and allele sequences discovered, and GBS has proven to be the most efficient method to evaluate genetic diversity of grain amaranth as well as to validate the phylogeny of the genus [15–17]. This genome assembly was used as a reference genome for an annotation framework and gene discovery of MYB-like transcription factor genes that regulate the betalain red pigment pathway, which gives rise to stem and seed colour variations [18] through traditional bi-parental mapping [14] and now through genome-wide association studies (GWAS) [19]. More recently, this plainsman reference genome together with low-coverage PacBio reads and the contigs of amaranth draft genome [20] were used to assemble *A*. *hypochondriacus* (A.hyp_K_white), a landrace cultivated in India [21]. This assembly offers a better reference genome for the improvement of grain and vegetable amaranth crops in South Asia as it is genetically closer to most landraces and accessions originated from India and South Asia.

Nevertheless, vegetable species of amaranth have been less studied by molecular means than pseudo-cereal grain amaranths and weed species, especially when both are phylogenically related and the domestication events separating them have been revealed [15, 19, 22, 23]. Limited knowledge of the genetic diversity in these leafy vegetable amaranth species and the lack of availability of suitable molecular markers hamper breeding efforts. Cultivar development and improvement relies on access to a well characterised, genetically diverse pool of material and so a comprehensive knowledge of these genetic relationships is essential. To date, there is only one molecular study that exploits a large number of *A*. *tricolor* accessions using simple sequence repeat (SSR) and *matK* protein-coding chloroplast gene, which concluded that the genetic diversity in Vietnamese amaranths was established by dispersal events mainly from East Asia and adaptation to local environments [24]. While the amaranth marker studies have been useful for evolutionary and phylogenetic studies, further germplasm characterization and marker validation is needed.

GBS offers a number of potential advantages to SSR markers; it is more practical, inexpensive and has driven genotyping to be applied for non-model organisms [25, 26]. DArTSeq^TM^ technology based on GBS methods is a platform developed by Diversity Arrays Technology Pty Ltd. (Canberra, Australia) for high-throughput genotyping via an intelligent selection of genome fraction by targeting active genes and low copy DNA areas [27, 28]. This present study is the first to utilize the DArTSeq platforms in amaranth to determine the genetic relationships and population structure between 188 amaranth accessions from 18 agronomically important vegetable, grain, and weedy species. This study also aimed to investigate the genetic relationship among a numerically larger group of *A*. *tricolor* accessions. The development of SNP markers from this panel has allowed a GWAS analysis on morphological traits such as shape, size, and colour of the leaf, stem, and inflorescence. These traits are fast and easy to assess for direct use by farmers and are of great help to plant breeders when selecting potential parental lines [12, 29]. This will facilitate understanding of the genetic bases and dissection of complex genes controlling economic traits such as drought tolerance and provides useful information on the degree of genetic variation and its correlations with agronomic traits.

## Materials and methods

### Plant materials, growing conditions and morphological assessment

A total of 188 amaranth accessions, comprising 18 species originating worldwide were used for genetic diversity analysis. Out of 188 accessions, 131 accessions were obtained from the World Vegetable Center Genebank, Taiwan (AVRDC), 52 accessions from the United State Department of Agriculture Genebank (USDA) and five commercial varieties were included as checks, of which three African varieties from East-West Seed, Thailand, were included and two local varieties from Serbajadi Gardening, Malaysia (Table 1).

**Table 1:** List of 188 amaranth accessions and their morphological traits observed under shade-house conditions.

Entry	Accessions	ID	Species	Origin country	Germplasm	Growth habit	Branching index	Stem colour	Leaf colour	Petiole colour	Inflorescence colour	Leaf shape	Leaf margin	Terminal inflorescence shape	Terminal inflorescence attitude
**1**	AV ATR	VI044435	Atropurperus	Indonesia	AVRDC	Erect	Along the stem	Pink	Normal green	Green	Green	Lanceolate	Entire	Short branches	Erect
**2**	AV GRA	VI036225	Graecizans	Hungary	AVRDC	Erect	Along the stem	Purple/Pink	Normal green	White	Pink	Lanceolate	Entire	Other	Erect
**3**	AV GRA SIL	VI044403	Graecizans ssp	India	AVRDC	Erect	Many branches	Purple/Pink	Normal green	Pink/Green	Pink	Cuneate	Entire	Other	Erect
**4**	AV GRA ASC	VI044388	Graecizans ssp	India	AVRDC	Erect	Many branches	Purple/Pink	Normal green	Pink/Green	Green	Obovate	Undulate	Other	Erect
**5**	AV MAN	VI044427	Mantegazzianus	USA	AVRDC	Erect	Few branches	Green	Normal green	Green	Yellow	Lanceolate	Entire	Club-shaped	Erect
**6**	AV BLITO	VI036227	Blitoides	Hungary	AVRDC	Prostate	Many branches	Purple/Pink	Normal green	Green	Green	Cuneate	Entire	Other	Erect
**7**	AV LEU	VI044445	Leucocarpus	India	AVRDC	Erect	Along the stem	Green	Normal green	Green	Green	Elliptical	Entire	Spike (dense)	Erect
**8**	AV PAL	VI044473	Palmeri	Senegal	AVRDC	Erect	Few branches	Purple/Pink	Normal green	Green	Green	Ovatainate	Entire	Other	Erect
**9**	AV RET 1	VI048310	Retroflexus	Viet Nam	AVRDC	Erect	Few branches	Purple/Pink	Margin/Vein	Purple	Red	Ovatainate	Entire	Spike (dense)	Erect
**10**	AV RET 2	VI048311	Retroflexus	Viet Nam	AVRDC	Erect	Along the stem	Green	Normal green	Green	Green	Lanceolate	Entire	Spike (dense)	Erect
**11**	AV RET 3	VI033461	Retroflexus	Venezuela	AVRDC	Erect	Many branches	Purple/Pink	chlorotic stripe	Green	Green	Rhombic	Undulate	Spike (dense)	Erect
**12**	AV RET 4	VI048391	Retroflexus	Viet Nam	AVRDC	Erect	Along the stem	Purple/Pink	Central spot	Purple	Red	Rhombic	Entire	Spike (dense)	Erect
**13**	US RET 1	Ames 26236	Retroflexus	China	USDA	Erect	Along the stem	Pink	Normal green	Green	Green	Elliptical	Entire	Long branches	Erect
**14**	AV SPI 1	VI044410	Spinosus	Puerto Rico	AVRDC	Erect	Along the stem	Purple/Pink	Normal green	Green	Green	Lanceolate	Entire	Short branches	Drooping
**15**	AV SPI 4	VI040944	Spinosus	Thailand	AVRDC	Erect	Many branches	Green	Normal green	Green	Green	Rhombic	Entire	Short branches	Erect
**16**	AV SPI 5	VI048723	Spinosus	Thailand	AVRDC	Erect	Along the stem	Green	One stripe	Green	Green	Rhombic	Undulate	Short branches	Erect
**17**	AV SPI 6	VI046123	Spinosus	Laos	AVRDC	Erect	Many branches	Purple/Pink	Normal green	Green	Green	Lanceolate	Undulate	Spike (dense)	Erect
**18**	AV SP 1	VI050253	Sp	Taiwan	AVRDC	Erect	Many branches	Green	Others	Green	Green	Rhombic	Undulate	Other	Erect
**19**	AV SP 2	VI049530	Sp	Thailand	AVRDC	Erect	Along the stem	Purple/Pink	Normal green	Green	Other	Elliptical	Entire	Short branches	Erect
**20**	AV SP 3	VI054799	Sp	Laos	AVRDC	Erect	Along the stem	Green	Normal green	Green	Green	Cuneate	Undulate	Short branches	Erect
**21**	AV SP 4	VI033471	Sp	Malaysia	AVRDC	Erect	Many branches	Green	Normal green	Green	Green	Lanceolate	Entire	Other	Erect
**22**	AV SP 5	VI044448	Sp	India	AVRDC	Erect	Many branches	Purple	Margin/Vein	Purple	Green/Red	Rhombic	Undulate	Other	Erect
**23**	AV SP 6	VI056563	Sp	Bangladesh	AVRDC	Erect	Many branches	Purple/Pink	Entire lamina	Dark purple	Red	Rhombic	Entire	Spike (dense)	Drooping
**24**	AV SP 7	VI056560	Sp	Bangladesh	AVRDC	Erect	Many branches	Green	Dark green	Green	Green	Rhombic	Entire	Spike (Dense)	Drooping
**25**	AV VIR 1	VI049893	Viridis	Thailand	AVRDC	Prostate	Many branches	Purple/Pink	Normal green	Green	Pink	Rhombic	Entire	Short branches	Erect
**26**	AV VIR 4	VI049001	Viridis	Thailand	AVRDC	Erect	Along the stem	Purple/Pink	Normal green	Purple	Green	Cuneate	Entire	Short branches	Erect
**27**	AV VIR 6	VI048697	Viridis	Thailand	AVRDC	Erect	Many branches	Purple/Pink	Normal green	Green	Pink	Ovatainate	Entire	Short branches	Erect
**28**	AV VIR 9	VI055027	Viridis	Malaysia	AVRDC	Erect	Few branches	Green	Normal green	Green	Green	Ovatainate	Undulate	Short branches	Erect
**29**	AV VIR 12	VI046127	Viridis	Laos	AVRDC	Erect	Along the stem	Purple/Pink	Normal green	Purple	Pink	Ovatainate	Entire	Short branches	Drooping
**30**	AV VIR 14	VI044432	Viridis	Indonesia	AVRDC	Erect	Along the stem	Purple/Pink	Normal green	Green	Other	Rhombic	Entire	Short branches	Drooping
**31**	AV CRU 1	VI036230	Cruentus	Austria	AVRDC	Erect	Along the stem	Purple/Pink	Normal green	Pink	Green	Elliptical	Entire	Long branches	Erect
**32**	AV CRU 2	VI044366	Cruentus	Ethiopia	AVRDC	Erect	Along the stem	Green	Normal green	Green	Green	Lanceolate	Undulate	Short branches	Erect
**33**	AV CRU 3	VI036231	Cruentus	Austria	AVRDC	Erect	Along the stem	Purple/Pink	Margin/Vein	Pink	Green/Red	Ovatainate	Entire	Short branches	Erect
**34**	AV CRU 5	VI044453	Cruentus	Mexico	AVRDC	Erect	Few branches	Purple/Pink	chlorotic stripe	Green	Yellow	Ovatainate	Undulate	Club-shaped	Erect
**35**	AV CRU 6	VI050473	Cruentus	Sudan	AVRDC	Erect	Many branches	Purple/Pink	Margin/Vein	Purple	Pink	Ovatainate	Entire	Short branches	Erect
**36**	AV CRU 12	VI044457	Cruentus	Zimbabwe	AVRDC	Erect	Along the stem	Green	Normal green	Green	Green	Lanceolate	Entire	Short branches	Erect
**37**	AV CRU 14	VI033487	Cruentus	Malaysia	AVRDC	Erect	Along the stem	Green	Central spot	Purple	Pink	Other	Undulate	Short branches	Erect
**38**	AV CRU 15	VI044449	Cruentus	Guatemala	AVRDC	Erect	Along the stem	Green	Normal green	Green	Green	Lanceolate	Undulate	Club-shaped	Erect
**39**	AV HYB 1	VI044419	Hybridus	USA	AVRDC	Erect	Few branches	Purple/Pink	Normal green	Green	Green	Rhombic	Undulate	Spike (dense)	Erect
**40**	AV HYB 2	VI044421	Hybridus	USA	AVRDC	Prostate	Few branches	Purple/Pink	Normal green	Green	Green	Cuneate	Undulate	Spike (dense)	Erect
**41**	AV HYB 3	VI051004	Hybridus	Kenya	AVRDC	Erect	Along the stem	Purple/Pink	Normal green	Purple	Green	Lanceolate	Entire	Spike (dense)	Drooping
**42**	US HYB 2	PI 641052	Hybridus	Nigeria	USDA	Erect	Along the stem	Purple	Entire lamina	Purple	Red	Elliptical	Entire	Spike (dense)	Erect
**43**	AV GRA 1	VI056002	Gracilis	Cambodia	AVRDC	Erect	Along the stem	Purple	Central spot	Green	Green	Ovatainate	Undulate	Spike (dense)	Drooping
**44**	EW CRU	#20866	Cruentus	Tanzania	E-WEST	Erect	Along the stem	Green	Normal green	Green	Green	Ovatainate	Undulate	Club-shaped	Erect
**45**	AV HYP 2	VI044454	Hypochondriacus	Mexico	AVRDC	Erect	Few branches	Purple/Pink	Normal green	Green	Green	Ovate	Entire	Long branches	Erect
**46**	AV HYP 3	VI044414	Hypochondriacus	India	AVRDC	Erect	Few branches	Green	Normal green	Green	Pink	Elliptical	Undulate	Long branches	Erect
**47**	AV HYP 5	VI036229	Hypochondriacus	Hungary	AVRDC	Erect	Along the stem	Purple/Pink	Margin/Vein	Pink	Pink	Lanceolate	Entire	Long branches	Drooping
**48**	AV HYP 6	VI044479	Hypochondriacus	Nepal	AVRDC	Erect	Few branches	Purple/Pink	Central spot	Green	Red	Lanceolate	Undulate	Long branches	Erect
**49**	AV HYP 10	VI044365-A	Hypochondriacus	Ghana	AVRDC	Erect	Along the stem	Green	Normal green	Green	Green	Lanceolate	Entire	Spike (dense)	Erect
**50**	AV HYP 13	VI047551	Hypochondriacus	Viet Nam	AVRDC	Erect	Along the stem	Purple/Pink	Entire lamina	Dark purple	Red	Lanceolate	Entire	Spike (Dense)	Erect
**51**	AV HYP 14	VI033462-A	Hypochondriacus	Ecuador	AVRDC	Erect	Few branches	Purple/Pink	Margin/Vein	Purple/Pink	Red	Elliptical	Undulate	Long branches	Drooping
**52**	AV HYP 16	VI044395	Hypochondriacus	Afghanistan	AVRDC	Erect	Many branches	Purple/Pink	Margin/Vein	Purple/Pink	Pink	Ovatainate	Entire	Spike (Dense)	Drooping
**53**	AV BLI 1	VI044404	Blitum cvg alecereus	India	AVRDC	Erect	Few branches	Purple/Pink	Normal green	Green	Green	Obovate	Undulate	Spike (dense)	Erect
**54**	AV BLI 3	VI055755	Blitum cvg alecereus	Laos	AVRDC	Erect	Along the stem	Green	Normal green	White	Green	Elliptical	Undulate	Spike (dense)	Erect
**55**	AV BLI 4	VI055121	Blitum cvg alecereus	Malaysia	AVRDC	Erect	Few branches	Purple/Pink	Normal green	Dark purple	Red	Elliptical	Entire	Spike (dense)	Erect
**56**	AV BLI 7	VI049036	Blitum	Thailand	AVRDC	Erect	Many branches	White	Normal green	Pink/Green	Green	Ovatainate	Undulate	Long branches	Erect
**57**	AV BLI 10	VI044447	Blitum	Korea	AVRDC	Prostate	Along the stem	Purple/Pink	Central spot	Green	Green	Ovatainate	Entire	Short branches	Erect
**58**	AV BLI 12	VI044423	Blitum	India	AVRDC	Erect	Many branches	Purple/Pink	Entire lamina	Purple	Red	Cuneate	Entire	Other	Erect
**59**	AV BLI 13	VI056127	Blitum	Cambodia	AVRDC	Erect	Along the stem	White	chlorotic stripe	White	Green	Ovatainate	Undulate	Short branches	Drooping
**60**	AV THU 1	VI050456	Thunbergii	Unknown	AVRDC	Erect	Few branches	Purple/Pink	Entire lamina	Purple	Red	Rhombic	Undulate	Short branches	Erect
**61**	AV THU 2	VI050467	Thunbergii	Unknown	AVRDC	Erect	Along the stem	Purple/Pink	Entire lamina	Purple	Pink	Ovatainate	Undulate	Spike (dense)	Erect
**62**	AV THU 3	VI050468	Thunbergii	Unknown	AVRDC	Erect	Along the stem	Purple/Pink	Margin/Vein	Purple	Green	Lanceolate	Undulate	Spike (dense)	Erect
**63**	AV DUB 1	VI047576	Dubius	Viet Nam	AVRDC	Erect	Along the stem	Green	Central spot	Green	Green	Ovatainate	Entire	Spike (Dense)	Erect
**64**	AV DUB 2	VI047537	Dubius	Viet Nam	AVRDC	Erect	Many branches	Pink/Green	Central spot	Pink/Green	Green	Ovatainate	Entire	Spike (Dense)	Erect
**65**	AV DUB 6	VI048985	Dubius	Thailand	AVRDC	Erect	Along the stem	Pink	Normal green	Pink	Pink	Cuneate	Undulate	Short branches	Erect
**66**	AV DUB 7	VI050464	Dubius	Tanzania	AVRDC	Erect	Along the stem	Purple/Pink	Normal green	Purple	Green	Elliptical	Undulate	Spike (Dense)	Erect
**67**	AV DUB 13	VI044377	Dubius	Surinam	AVRDC	Erect	Along the stem	Pink	Normal green	Pink	Green	Ovatainate	Undulate	Spike (Dense)	Erect
**68**	AV DUB 15	VI057160	Dubius	Cambodia	AVRDC	Erect	Along the stem	Purple/Pink	Central spot	Purple	Green	Lanceolate	Undulate	Spike (Dense)	Drooping
**69**	AV TRI 1	VI038237	Tricolor	Bangladesh	AVRDC	Erect	Many branches	Purple/Pink	Entire lamina	Dark purple	Red	Rhombic	Undulate	Short branches	Erect
**70**	AV TRI 2	VI055356	Tricolor	Bangladesh	AVRDC	Erect	No branches	Purple/Pink	chlorotic stripe	Purple	Green	Rhombic	Undulate	Short branches	Drooping
**71**	AV TRI 3	VI055353	Tricolor	Bangladesh	AVRDC	Erect	Along the stem	Purple/Pink	chlorotic stripe	Purple	Green/Red	Elliptical	Undulate	Short branches	Drooping
**72**	AV TRI 4	VI055350	Tricolor	Bangladesh	AVRDC	Erect	Many branches	Purple/Pink	Entire lamina	Purple	Red	Ovatainate	Undulate	Long branches	Erect
**73**	AV TRI 5	VI048269	Tricolor	Bangladesh	AVRDC	Erect	Along the stem	Purple/Pink	Central spot	Purple	Red	Ovatainate	Undulate	Spike (dense)	Erect
**74**	AV TRI 6	VI048233-A	Tricolor	Bangladesh	AVRDC	Erect	Along the stem	Green	Normal green	Green	Green	Ovatainate	Entire	Spike (dense)	Erect
**75**	AV TRI 7	VI048200	Tricolor	Bangladesh	AVRDC	Erect	Along the stem	Purple/Pink	Entire lamina	Purple	Green/Red	Ovatainate	Entire	Short branches	Erect
**76**	AV TRI 8	VI048146	Tricolor	Bangladesh	AVRDC	Erect	Along the stem	Purple/Pink	Entire lamina	Purple	Green/Red	Ovatainate	Entire	Short branches	Erect
**77**	AV TRI 9	VI048089	Tricolor	Bangladesh	AVRDC	Erect	Along the stem	Purple/Pink	Entire lamina	Purple	Green/Red	Ovatainate	Entire	Spike (dense)	Erect
**78**	AV TRI 10	VI047848	Tricolor	Bangladesh	AVRDC	Erect	Along the stem	Purple/Pink	Entire lamina	Purple	Green/Red	Ovatainate	Entire	Short branches	Erect
**79**	AV TRI 11	VI047795	Tricolor	Bangladesh	AVRDC	Erect	Few branches	Purple/Pink	Entire lamina	Purple	Green/Red	Rhombic	Entire	Short branches	Erect
**80**	AV TRI 12	VI044420	Tricolor	China	AVRDC	Erect	Few branches	Purple/Pink	Central spot	Green	Green	Cuneate	Entire	Spike (dense)	Erect
**81**	EW TRI Thida	Thida	Tricolor	Malaysia	E-WEST	Erect	Along the stem	Green	Normal green	Green	Green	Ovatainate	Entire	Spike (dense)	Erect
**82**	EW TRI Zeya	Zeya	Tricolor	Malaysia	E-WEST	Erect	Along the stem	Green	Central spot	Green	Green	Ovatainate	Entire	Spike (dense)	Erect
**83**	AV TRI 15	VI042983	Tricolor	Indonesia	AVRDC	Erect	Along the stem	Green	Normal green	Green	Green	Rhombic	Entire	Spike (dense)	Erect
**84**	AV TRI 16	VI047439	Tricolor	India	AVRDC	Erect	Along the stem	Purple/Pink	Central spot	Purple	Green	Ovatainate	Undulate	Other	Erect
**85**	AV TRI 17	VI048528	Tricolor	Japan	AVRDC	Erect	Many branches	Green	Others	Green	Green	Rhombic	Undulate	Short branches	Erect
**86**	AV TRI 18	VI044446	Tricolor	India	AVRDC	Erect	Along the stem	Green	Two stripes	Green	Green	Lanceolate	Undulate	Short branches	Erect
**87**	AV TRI 19	VI044443	Tricolor	India	AVRDC	Erect	Few branches	Green	Normal green	White	Yellow	Rhombic	Entire	Club-shaped	Erect
**88**	AV TRI 20	VI043725	Tricolor	Malaysia	AVRDC	Erect	Along the stem	Purple/Pink	Central spot	Purple	Red	Other	Undulate	Short branches	Erect
**89**	AV TRI 21	VI043724	Tricolor	Malaysia	AVRDC	Erect	Along the stem	Purple/Pink	Central spot	Purple	Green	Ovatainate	Entire	Long branches	Drooping
**90**	AV TRI 22	VI044438-A	Tricolor	Nigeria	AVRDC	Erect	Few branches	Green	Normal green	Green	Green	Rhombic	Undulate	Long branches	Erect
**91**	AV TRI 23	VI055809	Tricolor	Laos	AVRDC	Erect	Along the stem	Green	Central spot	Green	Green	Rhombic	Undulate	Long branches	Erect
**92**	AV TRI 24	VI044396-A	Tricolor	Pakistan	AVRDC	Erect	Along the stem	Green	Normal green	Green	Green	Mixture	Entire/Undulate	Long branches	Erect
**93**	AV TRI 25	VI049129	Tricolor	Thailand	AVRDC	Erect	Along the stem	Purple/Pink	Central spot	Green	Green	Elliptical	Undulate	Long branches	Drooping
**94**	AV TRI 26	VI049006	Tricolor	Thailand	AVRDC	Erect	Few branches	Purple/Pink	Central spot	Green	Green	Ovatainate	Entire	Long branches	Erect
**95**	AV TRI 27	VI049004	Tricolor	Thailand	AVRDC	Erect	Many branches	Green	Normal green	Green	Green	Lanceolate	Undulate	Short branches	Erect
**96**	AV TRI 28	VI044389	Tricolor	Turkey	AVRDC	Erect	No branches	Purple/Pink	Normal green	Green	Green	Ovatainate	Entire	Spike (dense)	Erect
**97**	AV TRI 29	VI044470	Tricolor	USA	AVRDC	Erect	Few branches	Green	Normal green	Green	Green	Lanceolate	Undulate	Spike (dense)	Erect
**98**	AV TRI 30	VI047747	Tricolor	Viet Nam	AVRDC	Erect	Along the stem	Green	chlorotic stripe	Green	Green	Rhombic	Undulate	Spike (dense)	Drooping
**99**	AV TRI 31	VI050615-A	Tricolor	Viet Nam	AVRDC	Erect	Along the stem	Purple/Pink	Entire lamina	Dark purple	Red	Ovatainate	Undulate	Spike (dense)	Erect
**100**	AV TRI 32	VI050613	Tricolor	Viet Nam	AVRDC	Erect	Many branches	Green	Normal green	Green	Green	Ovatainate	Entire	Short branches	Erect
**101**	AV TRI 33	VI050610-A	Tricolor	Viet Nam	AVRDC	Erect	Along the stem	Green	Normal green	Green	Green	Lanceolate	Undulate	Short branches	Erect
**102**	AV TRI 34	VI050609-A	Tricolor	Viet Nam	AVRDC	Erect	Many branches	Purple/Pink	Central spot	Purple	Green	Rhombic	Entire	Short branches	Erect
**103**	AV TRI 35	VI047603	Tricolor	Viet Nam	AVRDC	Erect	Along the stem	Purple/Pink	Entire lamina	Purple	Pink	Ovatainate	Entire	Spike (dense)	Erect
**104**	Local PR	var. BBS014	Tricolor	Unknown	LOCAL	Erect	Along the stem	Purple/Pink	Entire lamina	Purple	Red	Ovatainate	Entire	Short branches	Erect
**105**	AV TRI 37	VI054536	Tricolor	Taiwan	AVRDC	Erect	Few branches	Pink	Central spot	Green	Green	Ovatainate	Undulate	Long branches	Drooping
**106**	AV TRI 38	VI050214	Tricolor	Taiwan	AVRDC	Erect	Along the stem	Pink	Central spot	Green	Green	Elliptical	Entire	Long branches	Erect
**107**	AV TRI 39	VI054572	Tricolor	Philippines	AVRDC	Erect	Many branches	Purple/Pink	Entire lamina	Dark purple	Red	Rhombic	Undulate	Long branches	Drooping
**108**	AV TRI 40	VI054571	Tricolor	Philippines	AVRDC	Erect	Along the stem	Purple/Pink	Entire lamina	Dark purple	Red	Ovatainate	Undulate	Long branches	Erect
**109**	AV TRI 41	VI044450	Tricolor	Papua New Guinea	AVRDC	Erect	Along the stem	Green	Spotted purple	Purple	Green	Lanceolate	Undulate	Other	Erect
**110**	AV TRI 42	VI044407	Tricolor	Papua New Guinea	AVRDC	Erect	Along the stem	Green	Spotted purple	Purple	Green	Elliptical	Entire	Other	Other
**111**	AV TRI 43	VI048301	Tricolor	Bangladesh	AVRDC	Erect	Along the stem	Purple	Entire lamina	Dark purple	Red	Ovatainate	Undulate	Short branches	Drooping
**112**	AV TRI 44	VI048286	Tricolor	Bangladesh	AVRDC	Erect	Along the stem	Purple/Pink	Margin/Vein	Pink/Green	Other	Mixture	Entire	Short branches	Erect
**113**	AV TRI 45	VI048021	Tricolor	Bangladesh	AVRDC	Erect	Few branches	Purple	Entire lamina	Purple	Red	Rhombic	Entire	Short branches	Drooping
**114**	AV TRI 46	VI047929	Tricolor	Bangladesh	AVRDC	Erect	Along the stem	Purple/Pink	Entire lamina	Purple	Green/Red	Ovatainate	Undulate	Short branches	Erect
**115**	AV TRI 47	VI047682	Tricolor	Bangladesh	AVRDC	Erect	Along the stem	Pink/Green	Margin/Vein	Green	Pink	Rhombic	Undulate	Spike (dense)	Drooping
**116**	AV TRI 48	VI047681	Tricolor	Bangladesh	AVRDC	Erect	Few branches	Green	Normal green	Green	Green	Ovatainate	Undulate	Spike (dense)	Erect
**117**	AV TRI 49	VI047504	Tricolor	Bangladesh	AVRDC	Erect	Few branches	Purple/Pink	Entire lamina	Dark purple	Red	Rhombic	Entire	Spike (dense)	Erect
**118**	AV TRI 50	VI047501	Tricolor	Bangladesh	AVRDC	Erect	Few branches	Purple	Entire lamina	Purple	Red	Mixture	Entire		Erect
**119**	AV TRI 51	VI057270	Tricolor	Cambodia	AVRDC	Erect	Few branches	Green	Normal green	Green	Green	Rhombic	Entire	Short branches	Drooping
**120**	AV TRI 52	VI056168	Tricolor	Cambodia	AVRDC	Erect	Few branches	Purple/Pink	Normal green	Green	Green	Other	Undulate	Short branches	Erect
**121**	AV TRI 53	VI042979	Tricolor	Indonesia	AVRDC	Erect	Few branches	Green	Normal green	Green	Green	Ovate	Undulate	Spike (dense)	Drooping
**122**	AV TRI 54	VI042978	Tricolor	Indonesia	AVRDC	Erect	Few branches	Green	Normal green	Green	Green	Ovate	Undulate	Spike (dense)	Erect
**123**	AV TRI 55	VI059413	Tricolor	India	AVRDC	Erect	Along the stem	Green	Normal green	Green	Green	Rhombic	Undulate	Other	Erect
**124**	AV TRI 56	VI058498	Tricolor	India	AVRDC	Erect	Along the stem	Purple/Pink	Margin/Vein	Purple	Pink	Lanceolate	Undulate	Other	Erect
**125**	AV TRI 57	VI044426	Tricolor	Malaysia	AVRDC	Erect	Few branches	Green	Normal green	Green	Green	Rhombic	Undulate	Other	Drooping
**126**	AV TRI 58	VI055139	Tricolor	Malaysia	AVRDC	Erect	Few branches	Purple/Pink	Central spot	Purple	Green	Rhombic	Entire	Other	Drooping
**127**	AV TRI 59	VI055062	Tricolor	Malaysia	AVRDC	Erect	Along the stem	Green	chlorotic stripe	Green	Green	Lanceolate	Entire	Other	Erect
**128**	AV TRI 60	VI033490	Tricolor	Malaysia	AVRDC	Erect	Along the stem	Purple/Pink	Central spot	Purple	Green	Rhombic	Undulate	Other	Erect
**129**	AV TRI 61	VI033480	Tricolor	Malaysia	AVRDC	Erect	Along the stem	Green	Normal green	Green	Green	Lanceolate	Undulate	Other	Erect
**130**	AV TRI 62	VI033474	Tricolor	Malaysia	AVRDC	Erect	Along the stem	Green	Others	Green	Green	Other	Undulate	Other	Erect
**131**	AV TRI 63	VI033473	Tricolor	Malaysia	AVRDC	Erect	Along the stem	Pink/Green	Central spot	Pink/Green	Green	Rhombic	Undulate	Short branches	Drooping
**132**	AV TRI 64	VI049005	Tricolor	Thailand	AVRDC	Erect	Along the stem	Green	chlorotic stripe	Green	Green	Other	Undulate	Other	Drooping
**133**	AV TRI 65	VI044379-A	Tricolor	USA	AVRDC	Erect	Few branches	Green	Normal green	Green	Green	Rhombic	Undulate	Other	Erect
**134**	AV TRI 66	VI047526-A	Tricolor	Viet Nam	AVRDC	Erect	Along the stem	Purple/Pink	Margin/Vein	Purple	Pink	Rhombic	Entire	Other	Erect
**135**	AV TRI 67	VI047387	Tricolor	Viet Nam	AVRDC	Erect	Along the stem	Green	Normal green	Green	Green	Lanceolate	Entire	Spike (dense)	Erect
**136**	AV TRI 68	VI050111	Tricolor	Taiwan	AVRDC	Erect	Along the stem	Green	Normal green	Green	Green	Lanceolate	Entire	Short branches	Erect
**137**	AV TRI 69	VI049431	Tricolor	Taiwan	AVRDC	Erect	Along the stem	Green	Normal green	Green	Green	Obovate	Undulate	Short branches	Erect
**138**	US TRI 1	Ames 5368	Tricolor	Bangladesh	USDA	Erect	Along the stem	Pink	Entire lamina	Purple	Red	Elliptical	Entire	Long branches	Erect
**139**	US TRI 2	Ames 29504	Tricolor	Brazil	USDA	Erect	Along the stem	Pink	Entire lamina	Purple	Red	Rhombic	Entire	Spike (dense)	Erect
**140**	US TRI 3	Ames 29505	Tricolor	Brazil	USDA	Erect	Along the stem	Pink	Entire lamina	Purple	Red	Rhombic	Entire	Spike (dense)	Drooping
**141**	US TRI 4	Ames 2017	Tricolor	China	USDA	Erect	Along the stem	Green	Normal green	Green	Green	Ovatainate	Entire	Spike (dense)	Erect
**142**	US TRI 5	PI 419121	Tricolor	China	USDA	Erect	Along the stem	Purple	Entire lamina	Purple	Red	Ovatainate	Entire	Short branches	Erect
**143**	US TRI 6	PI 478310	Tricolor	China	USDA	Erect	Along the stem	Purple/Pink	Mixture	Pink/Green	Green/Red	Ovatainate	Entire	Spike (dense)	Drooping
**144**	US TRI 7	Ames 2204	Tricolor	Hong Kong	USDA	Erect	Along the stem	Pink	Central spot	Green	Green	Ovatainate	Undulate	Short branches	Erect
**145**	US TRI 8	Ames 2205	Tricolor	Hong Kong	USDA	Erect	Along the stem	Green	Normal green	Green	Green	Ovatainate	Entire	Short branches	Erect
**146**	US TRI 9	Ames 2040	Tricolor	India	USDA	Erect	Along the stem	Green	Normal green	Green	Green	Ovatainate	Undulate	Short branches	Erect
**147**	US TRI 10	Ames 2145	Tricolor	India	USDA	Erect	Along the stem	Pink	Normal green	Green	Green	Ovate	Undulate	Short branches	Erect
**148**	US TRI 11	PI 669847	Tricolor	India	USDA	Erect	Along the stem	Purple	Entire lamina	Purple	Red	Ovatainate	Undulate	Short branches	Erect
**149**	US TRI 12	PI 674261	Tricolor	India	USDA	Erect	Along the stem	Purple	Entire lamina	Purple	Red	Ovatainate	Undulate	Short branches	Erect
**150**	US TRI 13	Ames 2039	Tricolor	Indonesia	USDA	Erect	Along the stem	Green	Normal green	Green	Green	Rhombic	Crenate	Short branches	Erect
**151**	US TRI 14	Ames 5354	Tricolor	Madagascar	USDA	Erect	Along the stem	Green	Basal area	Green	Green	Ovate	Entire	Long branches	Erect
**152**	US TRI 15	Ames 2029	Tricolor	Malaysia	USDA	Erect	Along the stem	Pink	Central spot	Purple	Green/Red	Elliptical	Entire	Spike (dense)	Erect
**153**	US TRI 16	Ames 29034	Tricolor	Malaysia	USDA	Erect	Along the stem	Others	Others	Others	Green	Rhombic	Undulate	Short branches	Erect
**154**	US TRI 17	Ames 5111	Tricolor	Papua New Guinea	USDA	Erect	Along the stem	Green	Normal green	Green	Green	Ovate	Entire	Spike (dense)	Erect
**155**	US TRI 18	PI 349553	Tricolor	Papua New Guinea	USDA	Erect	Along the stem	Green	Spotted purple	Purple	Green	Ovate	Crenate	Other	Other
**156**	US TRI 19	Ames 2199	Tricolor	Taiwan	USDA	Erect	Along the stem	Green	Central spot	Purple/Pink	Green	Ovatainate	Entire	Long branches	Erect
**157**	US TRI 20	Ames 2024	Tricolor	Thailand	USDA	Erect	Along the stem	Green	Normal green	Green	Green	Rhombic	Undulate	Long branches	Erect
**158**	US TRI 21	PI 607446	Tricolor	Thailand	USDA	Erect	Along the stem	Green	Normal green	White	Green	Ovatainate	Entire	Short branches	Erect
**159**	US TRI 22	PI 603897	Tricolor	USA	USDA	Erect	Along the stem	Purple	Entire lamina	Purple	Red	Elliptical	Entire	Other	Other
**160**	US TRI 23	PI 603898	Tricolor	USA	USDA	Erect	Along the stem	Purple	Margin/Vein	Purple	Red	Elliptical	Crenate	Other	Other
**161**	US TRI 24	PI 632237	Tricolor	USA	USDA	Erect	Along the stem	Purple	Central spot	Green	Green	Rhombic	Entire	Long branches	Erect
**162**	US TRI 25	Ames 5110	Tricolor	West Africa	USDA	Erect	Along the stem	Green	Normal green	Green	Green	Elliptical	Entire	Long branches	Erect
**163**	US TRI 26	Ames 1980	Tricolor	Zaire	USDA	Erect	Along the stem	Green	Normal green	Green	Green	Ovatainate	Entire	Long branches	Erect
**164**	US TRI 27	Ames 26209	Tricolor	China	USDA	Erect	Along the stem	Pink	Basal area	Green	Green	Rhombic	Entire	Spike (dense)	Erect
**165**	Local Red	var. BBS027	Tricolor	Malaysia	LOCAL	Erect	Along the stem	Green	Central spot	Green	Green	Ovatainate	Entire	Short branches	Erect
**166**	US TRI 29	Ames 26216	Tricolor	China	USDA	Erect	Along the stem	Purple	Entire lamina	Purple	Red	Lanceolate	Entire	Spike (dense)	Erect
**167**	US TRI 30	Ames 5102	Tricolor	Hong Kong	USDA	Erect	Along the stem	Purple/Pink	Central spot	Pink/Green	Green	Lanceolate	Entire	Other	Drooping
**168**	US TRI 31	Ames 5317	Tricolor	Hong Kong	USDA	Erect	Along the stem	Green	Normal green	Green	Green	Other	Entire	Other	Erect
**169**	US TRI 32	PI 674260	Tricolor	Hong Kong	USDA	Erect	Along the stem	Pink/Green	Central spot	Green	Green	Other	Entire	Spike (dense)	Erect
**170**	US TRI 33	Ames 2100	Tricolor	India	USDA	Erect	Along the stem	Purple	Entire lamina	Purple/Pink	Red	Lanceolate	Undulate	Spike (dense)	Erect
**171**	US TRI 34	Ames 2101	Tricolor	India	USDA	Erect	Along the stem	Purple/Pink	Margin/Vein	Pink	Red	Lanceolate	Entire	Short branches	Erect
**172**	US TRI 35	Ames 2102	Tricolor	India	USDA	Erect	Along the stem	Purple/Pink	Margin/Vein	Purple/Pink	Red	Rhombic	Entire	Short branches	Erect
**173**	US TRI 36	Ames 2119	Tricolor	India	USDA	Erect	Along the stem	Green	Normal green	Green	Green	Ovatainate	Entire	Other	Erect
**174**	US TRI 37	Ames 2120	Tricolor	India	USDA	Erect	Along the stem	Pink/Green	Margin/Vein	Pink	Green	Other	Undulate	Other	Erect
**175**	US TRI 38	Ames 2121	Tricolor	India	USDA	Erect	Along the stem	Pink	Normal green	Green	Green	Elliptical	Entire	Short branches	Erect
**176**	US TRI 39	Ames 2132	Tricolor	India	USDA	Erect	Along the stem	Purple	Margin/Vein	Purple	Red	Lanceolate	Entire	Short branches	Erect
**177**	US TRI 40	Ames 2134	Tricolor	India	USDA	Erect	Along the stem	Green	Normal green	Green	Green	Lanceolate	Entire	Short branches	Erect
**178**	US TRI 41	Ames 2135	Tricolor	India	USDA	Erect	Along the stem	Green	Normal green	Green	Green	Lanceolate	Entire	Short branches	Erect
**179**	US TRI 42	Ames 2138	Tricolor	India	USDA	Erect	Along the stem	Green	Normal green	Green	Green	Lanceolate	Entire	Short branches	Erect
**180**	US TRI 43	Ames 2223	Tricolor	India	USDA	Erect	Along the stem	Purple/Pink	Margin/Vein	Pink	Green	Lanceolate	Entire	Short branches	Erect
**181**	US TRI 44	Ames 2224	Tricolor	India	USDA	Erect	Along the stem	Pink/Green	Central spot	Pink/Green		Lanceolate	Entire	Short branches	Erect
**182**	US TRI 45	Ames 5117	Tricolor	Puerto Rico	USDA	Erect	Along the stem	Pink	Normal green	Pink	Green	Lanceolate	Entire	Other	Erect
**183**	US TRI 46	Ames 5118	Tricolor	Puerto Rico	USDA	Erect	Along the stem	Green	Normal green	Green	Green	Lanceolate	Entire	Short branches	Erect
**184**	US TRI 47	Ames 1993	Tricolor	Taiwan	USDA	Erect	Along the stem	Pink/Green	Central spot	Green	Green/Red	Ovatainate	Entire	Short branches	Erect
**185**	US TRI 48	Ames 1998	Tricolor	Taiwan	USDA	Erect	Along the stem	Green	Normal green	Green	Green	Other	Entire	Other	Erect
**186**	US TRI 49	Ames 5134	Tricolor	USA	USDA	Erect	Along the stem	Green	Normal green	Green	Green	Lanceolate	Entire	Long branches	Erect
**187**	US TRI 50	Ames 25153	Tricolor	USA	USDA	Erect	Along the stem	Pink/Green	Basal area	Green	Green	Elliptical	Entire	Short branches	Erect
**188**	US TRI 51	PI 633591	Tricolor	Unknown	USDA	Erect	Along the stem	Pink/Green	Basal area	Green	Green	Ovatainate	Entire	Other	Erect

A single plant of each accession was grown under shade-house conditions at University of Nottingham Malaysia (latitude 2.940°N, longitude 101.8740°E), with an average of 36°C daytime temperature, 28°C night temperature and 66% relative humidity. Plants were grown in a 16 x 12.5 x 14.5 cm plastic pot containing 2 kg black compost (Holland peat, Malaysia), irrigated daily to field capacity and at 3 weeks old, 3 g of 15N: 15P: 15K fertilizer was applied once to individual pots.

Ten qualitative traits including leaf, petiole and stem pigmentations, growth habit, branching index and, leaf shape and margin were recorded at 7 weeks post-emergence and terminal inflorescence color, shape and attitude were recorded when all accessions had fully set (at 11 weeks post emergence) using AVRDC descriptors (https://avrdc.org/seed/) (Table 1). Young leaf material was collected and snap frozen in liquid N_2_ and stored at -80°C for DNA analysis.

### DNA extraction and DArTSeq genotyping

Total genomic DNA of 188 amaranth accessions was isolated from young leaves using a Qiagen DNeasy plant DNA extraction kit (Qiagen, USA) and DNA quality and quantity was evaluated using a Nanodrop spectrophotometer (Thermo Scientific, USA). The DNA concentration was adjusted within the range of 50-100ng/μl. 2 μg of high molecular weight and good quality DNA per sample was sent to Diversity Arrays Technology Pty Ltd, Canberra, Australia for DArTSeq analysis.

In brief, DArTSeq technology relies on the combination of a complexity reduction method to enrich genomic representations, followed by next-generation sequencing by HiSeq2000 (Illumina, USA), as described by Kilian et al. [27]. In this study, a combination of a rare cutting methylation-sensitive restriction enzyme (RE) *PstI* with secondary frequently cutting RE *MseI* were selected to optimize the locus coverage, reproducibility and polymorphisms. The *PstI*-compatible adapter consists of the Illumina flow cell attachment sequence, sequencing primer and a ‘staggered’ of varying length barcode region. The reverse adapter consists of Illumina flow cell attachment region and *MseI* overhang sequence. The ligated fragments with both a *PstI* and *MseI* adapter were amplified via polymerase chain reaction (PCR) with a programme set to an initial denaturation step of 94°C for 1 min, followed by 30 cycles of denaturation at 94°C for 20 s, annealing at 58°C for 30 s and extension at 72°C for 45 s, before a final extension at 72°C for 7 min. Equimolar amounts of PCR products from each sample were combined followed by a single end sequencing of 77 cycles on an Illumina Hiseq2500. Twenty-four DNA samples were also genotyped in two technical replications to obtain the reproducibility of the marker data. The full SNP dataset is shown in S1 Table.

### Data analysis

#### SNP filtering

The SNP data generated from DArTSeq technology were first physically mapped to *Amaranthus hypochondriacus* genome v2.1 [14] using CLC Genomic Workbench v8 (Qiagen), based on match of aligned sequence tags against the reference genome, with 80% length and similarity fraction [29]. To investigate species-specific SNPs among 12 amaranth species (not including species with one representative), the amaranth species were manually examined for unique SNPs presence in the mapped SNP markers. Six species with the highest species-specific SNPs were subjected to a Venn diagram to visualize the SNP loci shared among the species. The Venn diagram of overlapping SNP loci was generated using the online program Van de Peer Lab (http://bioinformatics.psb.ugent.be/). Genetic diversity and population structure was carried out in two steps. First, all 188 amaranth accessions consisting of 18 species were analyzed together and second, a subset of 120 *A*. *tricolor* accessions were analyzed separately, aiming to explore the genetic distances and population structure among the *A*. *tricolor* populations, which were of primary interest. In each dataset, the mapped SNP markers were trimmed by removing SNPs with <97% reproducibility, <70% call rate and <0.05 polymorphic information content (PIC) and SNPs located on minor contigs that were not have been annotated. Individual accessions with >30% missing data and SNP loci with >30% missing data were removed. The most informative SNPs with minimum allele frequency (MAF) >0.05 imputed using TASSEL v5.2.52 software [30] were selected for further analysis.

Population structure was constructed using the structure-like population genetic analyses using R package LEA [31–33]. The number of populations was determined using cross-entropy criterion, based on the predictions of a fraction of masked genotypes (matrix completion) and on the cross-validation approach, with runs of eight values of K (K = 1:8). A distance matrix was generated using TASSEL v5.2.52 software which was used to conduct principal coordinate analysis (PCoA) and a phylogenetic tree based on UPGMA distance.

#### Genome-wide association study of morphological traits

GWAS was conducted on the observed ten morphological traits on the same SNP datasets used for genetic diversity analysis. A mixed linear model (MLM) was generated to determine the associations by using the Q-matrix from population structure analysis (R package LEA) and kinship (K) from centered IBS method via TASSEL v5.2.52 and marker trait association (MTA) was determined at P≤0.01 and P≤0.001. The Manhattan plots of–log(p-values) and the quantile-quantile plots (Q-Q) of expected vs observed p-values for SNP based genotype-phenotype associations were generated using TASSEL v5.2.52. The most significant flanking sequences of SNPs associated with the traits (P≤0.001) were queried against JBrowse Phytozome v13 database to obtain the putative biological functions.

## Results

### SNP marker discovery

**Fig 1:**
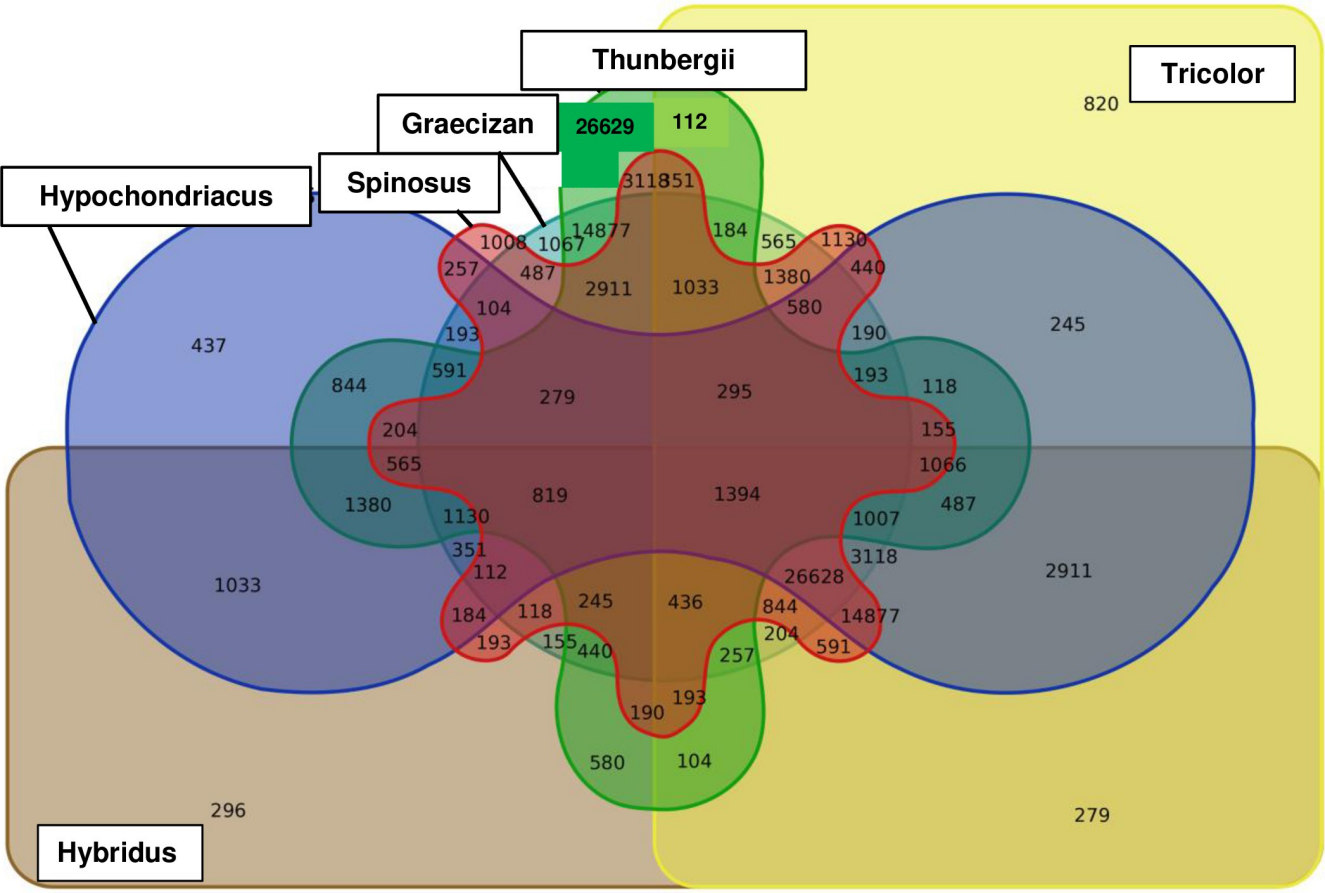
Venn diagram showing the presence, average and overlap of SNPs in the six largest amaranth species sets.

DArTSeq generated 74,306 polymorphic SNP reads from the 188 amaranth accessions of 18 species (S1 Table). Of these reads, 63,821 SNPs could be physically mapped to the *Amaranthus hypochondriacus* genome with an averaged of 100% reproducibility (max = 100%, min = 93%, median = 100%), 74% call rate (max = 100%, min = 19%, median = 74%) and 0.14 PIC (max = 0.50, min = 0, median = 0.09). The majority of the SNPs were an A/G or C/T transition mutation (62%) while the other 38% were A/C, A/T, C/G, and G/T transversion mutation. A Venn diagram of the six largest sets of amaranth species showed species-specific SNP loci, with *A*. *thunbergii* showed the highest number of unique SNPs (26,629), followed by *A*. *spinosus* (1,008), *A*. *graecizans* (1,067), *A*. *tricolor* (820), *A*. *hypochondriacus* (437) and *A*. *hybridus* (296) (Fig 1). There were only 1,394 polymorphic SNP shared by all six species group.

### Genetic diversity and population structure of two amaranth sets

First, for all 18 amaranth species, individual genotypes with >30% missing SNP data including *A*. *atropurperus* (AV-ATR), *A*. *blitoides* (AV-BLITO), and *A*. *spinosus* (AV-SPI 1, AV-SPI 5 and AV-SPI 6), *A*. *retroflexus* (US-RET 1) and *A*. *hybridus* (AV-HYB 3) were removed and a total of 8,668 SNPs remained for 16 amaranth species, comprised of 181 accessions, with an averaged of 100% reproducibility, 91% call rate, 0.28 PIC, 0.16 MAF, and 6.97% averaged missing value in SNP loci and 5% averaged missing value at the individual-level. Second, for 120 *A*. *tricolor* accessions, two individual accessions (AV-TRI 20 and AV-TRI 28) which contribute to 30% of the missing values were removed and a total of 9,789 SNPs remained for 118 *A*. *tricolor* accessions, with and averaged of 100% reproducibility, 78% call rate, 0.20 PIC, 0.07 MAF, and 2% averaged missing values in SNP loci and at individual-level. Both SNP datasets (from 16 amaranth species [181 accessions] and 118 *A*. *tricolor* accessions) shared 1346 SNPs identical markers.

**Fig 2:**
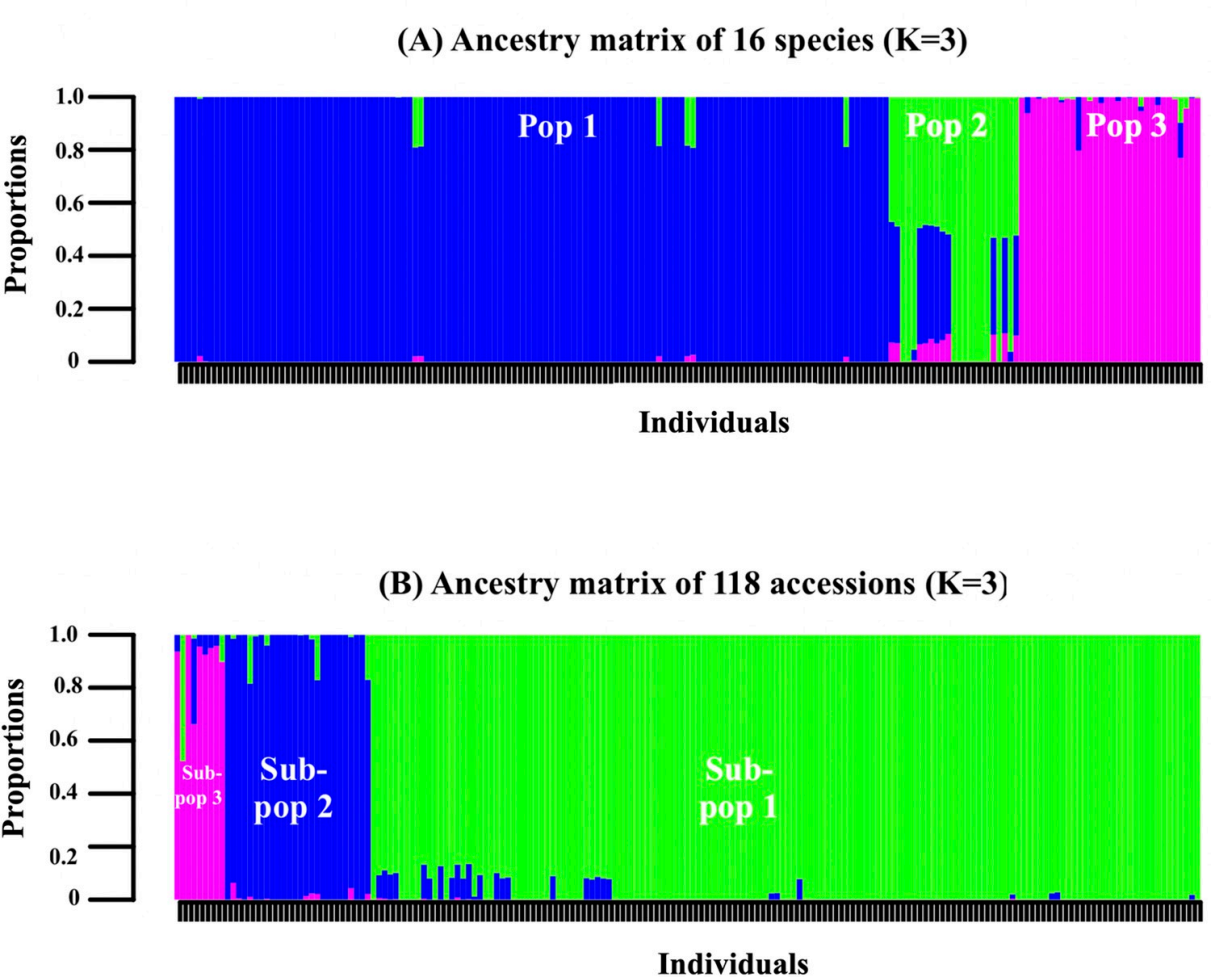
Population structure of (A) 16 amaranth species and (B) 118 *A*. *tricolor* accessions at K = 3, respectively. Each vertical bar represents a single accession and the length of each bar represents the proportion contributed by each sub-population. The group membership for each population structure is similar to the UPGMA dendogram.

**Fig 3:**
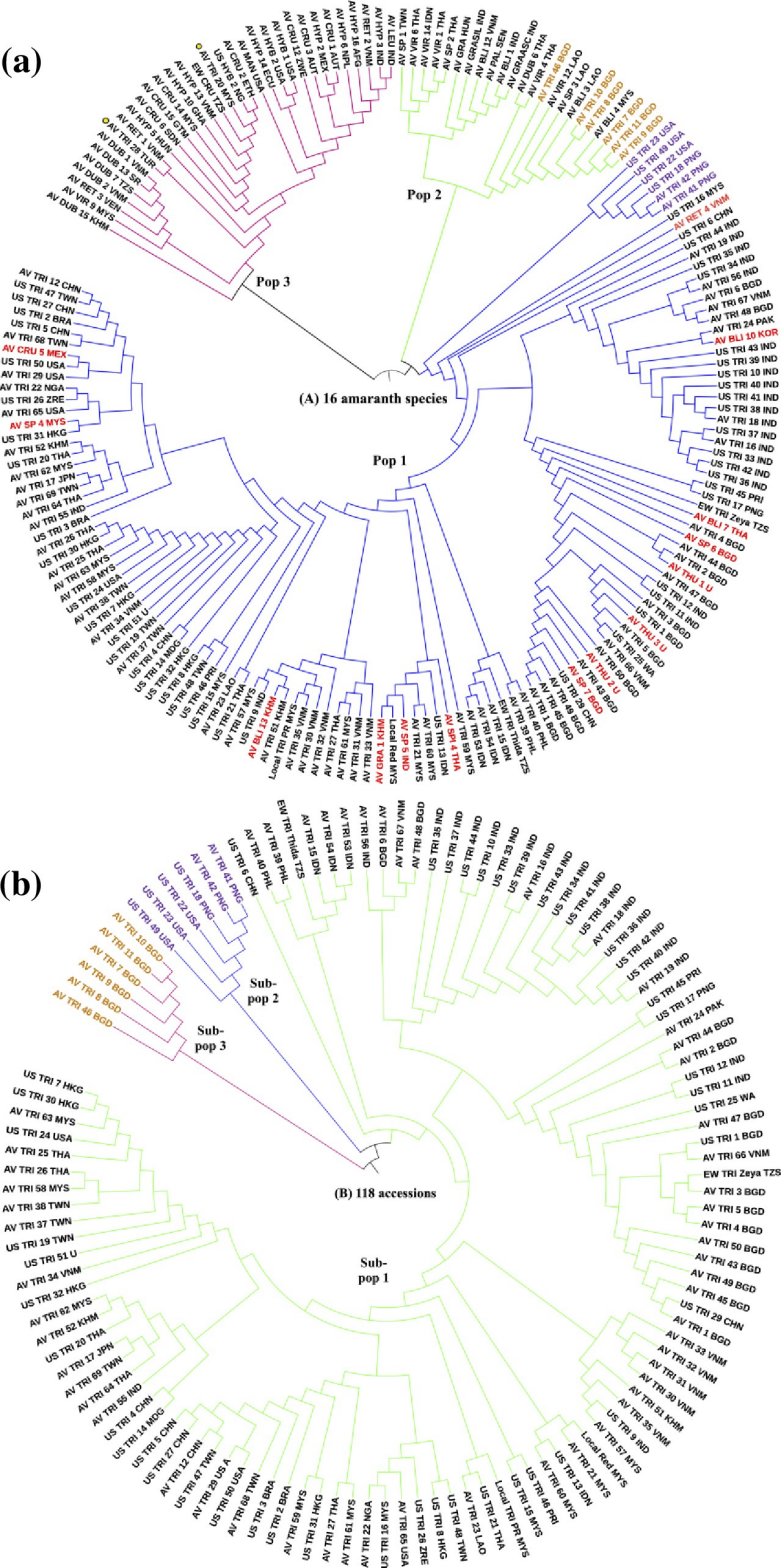
UPGMA phylogenetic tree of (A) 16 amaranth species and (B) 118 *A*. *tricolor* accessions. Yellow-dotted accessions were out-grouped *A*. *tricolor* and red-colored accessions were amaranth species that are closely related to most *A*. *tricolor*. Purple-colored and yellow-colored accessions were positioned in different clades of the second population structure as in (B).

**Fig 4:**
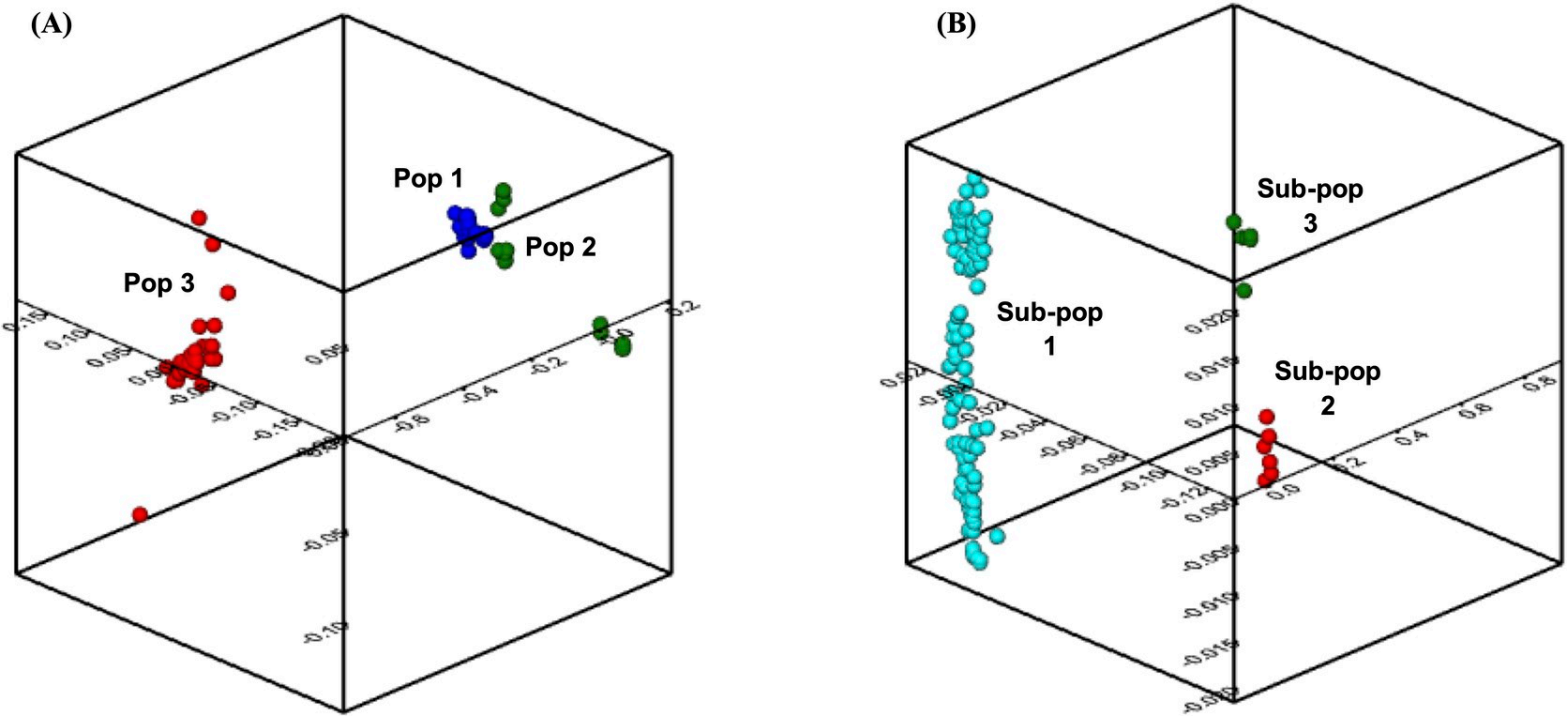
3D-plot principles coordinate analysis of (A) 16 amaranth species and (B) 118 *A*. *tricolor* accessions.

Population structure analysis demonstrated that the K-values of the 16 amaranth species dataset and the *A*. *tricolor* subset were K = 3 respectively, based on minimal cross-entropy (S1 Fig) and the Q-matrix is displayed in a bar plot representation (Fig 2A and 2B). Each vertical bar represents a single accession, and the length of each bar represents the proportion contributed by each sub-population (admixture) and the grouping of the populations are illustrated in UPGMA phylogenetic tree (Fig 3A and 3B). The PCoA demonstrates the genetic divergence of both marker datasets was consistent with the output of the population structure (Fig 4A and 4B).

The 16 amaranth species were grouped into three populations. The majority of *A*. *tricolor* accessions belonged to Pop 1, with the exception of six *A*. *tricolor* accessions which originated from Bangladesh and belonged to POP 2 (brown-colored accessions) while the two out-grouped *A*. *tricolor* accessions (AV-TRI 20 and AV-TRI 28) were separated into POP 3 (yellow-dotted colour accessions). The two grain-types amaranth species (*A*. *hypochondriacus* and *A*. *cruentus*) belonged to Pop 3 together with their putative progenitor (*A*. *hybridus*), with the exception of one *A*. *cruentus* accession (AV-CRU 5) which belongs to Pop 1. Other cultivated vegetable-type species such as *A*. *blitum*, *A*. *graecizan*, *A*. *sp* and *A*. *thunbergii* were closely related to *A*. *tricolor* in Pop 1 (red-colored accessions), although several accessions belonged to Pop 2. The weed-type species such as *A*. *retroflexus* and *A*. *viridis* were diverse between the three populations. The PCoA demonstrated that Pop 1 clustered tightly together depicting that little diversity may exist within the populations and closer to Pop 2 which may explain the inter-specific admixtures. Meanwhile Pop 2 and Pop 3 showed some dispersal and diversity within the populations.

The *A*. *tricolor* subset demonstrated that accessions were divided into three sub-populations. Sub-pop 1 was made up of 105 accessions from 12 countries of origin, Sub-Pop 2 comprised of seven accessions, of which three accessions were from Papua New Guinea and four accessions from USA, and Sub-pop 3 consists of six Bangladeshi accessions with distinct morphological traits (had branches along the stem, purple-pink stem color, purple leaf and petiole color, red-green inflorescence color and erect terminal inflorescence attitude) (Table 1). In comparison with the 16 amaranth species population structure, *A*. *tricolor* accessions that belong to Sub-pop 2 grouped together with the rest of *A*. *tricolor* accessions in Pop 1 (brown-colored accessions). Meanwhile, the six distinct Bangladeshi *A*. *tricolor* accessions of Sub-pop 3 remained separated from the rest of *A*. *tricolor* accessions similar to Pop 2. The PCoA displayed a clear division between the sub-populations and the overall population statistic calculated using a Monte-Carlo test revealed that there is an overall significant difference between the sub-populations (P = 0.002).

### SNP associations for morphological traits

GWAS identified 22 significant and “suggestive” MTAs on 16 chromosomes of 16 amaranth species that underline four morphological traits observed in branching index, inflorescence color, leaf shape (P≤0.01, P≤0.001) and leaf pigmentation (P≤0.01) (Table 2; S2 Table). At P≤0.001, four SNP markers were associated with branching index, six SNP markers associated with inflorescence color and two SNP markers associated with leaf shape. Meanwhile, 100 significant MTAs were generated from 118 *A*. *tricolor*, distributed among 16 chromosomes that underline four morphological traits observed in inflorescence color, and leaf, petiole and stem pigmentations (P≤0.01, P≤0.001) (Table 3, S2 Table). At P≤0.001, forty-five SNP markers were associated with leaf pigmentation, eight SNP markers associated with petiole pigmentation, four SNP markers associated with inflorescence color and two SNP markers associated with stem pigmentation.

**Table 2:** 12 MTAs (P≤0.001) of three morphological traits; branching index, inflorescence color and leaf shape in 16 amaranth species.

Trait	SNP Allele ID	Chr	SNP position (bp)	P	Marker R^2^	Transcript name	Protein homologs
Branching index	33457123|F|0–18:G>A-18:G>A	3	1229325	0.000	0.09588	-	
	33427832|F|0–36:A>T-36:A>T	14	1.1E+07	0.001	0.0854		
	33440918|F|0–66:G>T-66:G>T	5	8485720	0.001	0.08314		
	33416254|F|0–26:C>T-26:C>T	1	2988352	0.001	0.08504		
Inflorescence color	33414045|F|0–22:A>G-22:A>G	14	2E+07	0.000	0.13234	-	
	33416574|F|0–45:G>A-45:G>A	11	1.4E+07	0.000	0.14652	-	
	33420897|F|0–6:A>G-6:A>G	12	3726457	0.000	0.10482	AH018341-RA	FTIP1: FT-interacting protein 1 (*A*. *thaliana*)
	33430487|F|0–18:C>T-18:C>T	5	3281805	0.000	0.18777	-	
	33423475|F|0–8:C>G-8:C>G	2	2.2E+07	0.001	0.08268		
	33435567|F|0–16:T>C-16:T>C	16	7310907	0.001	0.08184		
Leaf shape	33459037|F|0–16:T>C-16:T>C	6	4618929	0.001	0.08407		
	33443436|F|0–14:G>A-14:G>A	6	4371684	0.001	0.08261		

**Table 3:** 58 MTAs (P≤0.001) of four morphological traits; inflorescence color, and leaf, petiole and stem pigmentations in 118 *A*. *tricolor* accessions.

Trait	SNP Allele ID	Chr	SNP position (bp)	P	Marker R^2^	Transcript name	Protein homologs
Inflorescence color	33421031|F|0–23:A>G-23:A>G	15	4478322	0.000	0.16	AH022173	Similar to MIEL1:E3 ubiquitin-protein ligase MIEL1 (*A*. *thaliana*)
	33435567|F|0–16:T>C-16:T>C	16	7310907	0.000	0.14		
	33416574|F|0–45:G>A-45:G>A	11	13603782	0.001	0.13		
	33439433|F|0–6:G>T-6:G>T	3	18420538	0.001	0.12	AH006016	Similar to MOT 1:MOLYBDATE TRANSPORTER 1 (*A*. *thaliana*)
Leaf pigmentation	33433818|F|0–31:C>A-31:C>A	14	19318346	0.000	0.23		
	33436368|F|0–13:T>C-13:T>C	2	19885054	0.000	0.21	AH003516	Similar to ATL: RING-H2 finger protein ATL78 (*A*. *thaliana*)
	33423765|F|0–13:C>A-13:C>A	2	19932945	0.000	0.21		
	33440367|F|0–5:G>A-5:G>A	2	32078565	0.000	0.20		
	33405774|F|0–47:T>C-47:T>C	2	27826039	0.000	0.20	AH003908	Similar to MRG1: Protein MRG1 (*A*. *thaliana*)
	33447836|F|0–29:C>T-29:C>T	8	4842922	0.000	0.19		
	33404573|F|0–8:T>C-8:T>C	10	21720524	0.000	0.18		
	33426216|F|0–17:T>C-17:T>C	11	14654529	0.000	0.18		
	33437340|F|0–31:A>G-31:A>G	10	21651122	0.000	0.18		
	33452287|F|0–24:G>A-24:G>A	4	261886	0.000	0.17		
	33415060|F|0–37:A>G-37:A>G	3	4287524	0.000	0.17		
	33416965|F|0–32:T>C-32:T>C	2	26027339	0.000	0.16		
	33448931|F|0–12:C>G-12:C>G	2	26006146	0.000	0.16		
	33437546|F|0–67:G>A-67:G>A	2	28467849	0.000	0.17		
	33415096|F|0–37:T>C-37:T>C	1	25218037	0.000	0.16		
	33415113|F|0–42:G>A-42:G>A	2	27277661	0.000	0.16		
	33405454|F|0–57:A>G-57:A>G	6	4790604	0.000	0.16		
	33445149|F|0–31:G>A-31:G>A	9	11064067	0.000	0.16		
	33443618|F|0–25:C>T-25:C>T	10	22131192	0.000	0.15		
	33423738|F|0–59:G>A-59:G>A	15	3381897	0.000	0.15		
	33439322|F|0–35:C>T-35:C>T	11	19694142	0.000	0.16		
	33458033|F|0–44:A>G-44:A>G	4	25006230	0.000	0.15		
	33423857|F|0–63:C>T-63:C>T	5	9104986	0.000	0.14		
	33440367|F|0–41:G>A-41:G>A	2	32078565	0.000	0.14		
	33448614|F|0–8:T>C-8:T>C	6	16703056	0.000	0.14		
	33427440|F|0–60:T>C-60:T>C	8	2505388	0.000	0.14		
	33453932|F|0–61:C>T-61:C>T	1	1128299	0.000	0.14		
	33417247|F|0–61:C>T-61:C>T	4	4275159	0.000	0.14		
	33403207|F|0–28:A>G-28:A>G	5	21853056	0.000	0.14		
	33426523|F|0–61:T>C-61:T>C	7	11525948	0.000	0.14		
	33410609|F|0–37:A>G-37:A>G	8	5819731	0.000	0.14		
	33430995|F|0–34:T>G-34:T>G	12	707152	0.000	0.14		
	33414569|F|0–44:A>G-44:A>G	13	15098294	0.000	0.14		
	33420689|F|0–33:G>A-33:G>A	3	4515916	0.000	0.14		
	33436571|F|0–8:A>G-8:A>G	4	14612733	0.000	0.15		
	33443221|F|0–41:G>A-41:G>A	10	21992096	0.000	0.13		
	33437028|F|0–10:C>T-10:C>T	4	20557141	0.000	0.13		
	33455993|F|0–25:T>C-25:T>C	4	20546481	0.000	0.13		
	33444523|F|0–46:G>A-46:G>A	10	22045821	0.001	0.14		
	33454339|F|0–18:C>T-18:C>T	8	16678507	0.001	0.13		
	33439286|F|0–20:G>A-20:G>A	16	6291635	0.001	0.17		
	33441539|F|0–51:C>T-51:C>T	4	27763448	0.001	0.12		
	33453929|F|0–59:A>C-59:A>C	4	10349586	0.001	0.12		
	33422968|F|0–36:C>A-36:C>A	2	20844422	0.001	0.13		
	33457404|F|0–39:C>T-39:C>T	13	2722366	0.001	0.13		
Petiole pigmentation	33432644|F|0–45:A>C-45:A>C	7	19047239	0.000	0.21	AH011978	Similar to DMS3: Protein DEFECTIVE IN MERISTEM SILENCING 3 (*A*.*thaliana*)
	33456924|F|0–8:T>C-8:T>C	7	19136116	0.000	0.15		
	33451216|F|0–61:C>T-61:C>T	8	9410249	0.000	0.14		
	33422926|F|0–18:G>C-18:G>C	10	20776062	0.001	0.14	AH016449	Similar to FPGS1: Folylpolyglutamate synthase (*A*. *thaliana*)
	33442805|F|0–17:T>C-17:T>C	11	11011768	0.001	0.12	AH017000	Similar to HT1:Serine/threonine-protein kinase HT1 (*A*. *thaliana*)
	33439355|F|0–33:G>A-33:G>A	3	11059585	0.001	0.12		
	33415765|F|0–49:T>C-49:T>C	7	18977396	0.001	0.12		
	33417264|F|0–60:C>G-60:C>G	7	18977331	0.001	0.12		
Stem pigmentation	33424999|F|0–57:T>C-57:T>C	3	8553631	0.000	0.20	AH005492	Similar to At1g68200: Zinc finger CCCH domain-containing protein 15 (*A*. *thaliana*)

**Fig 5:**
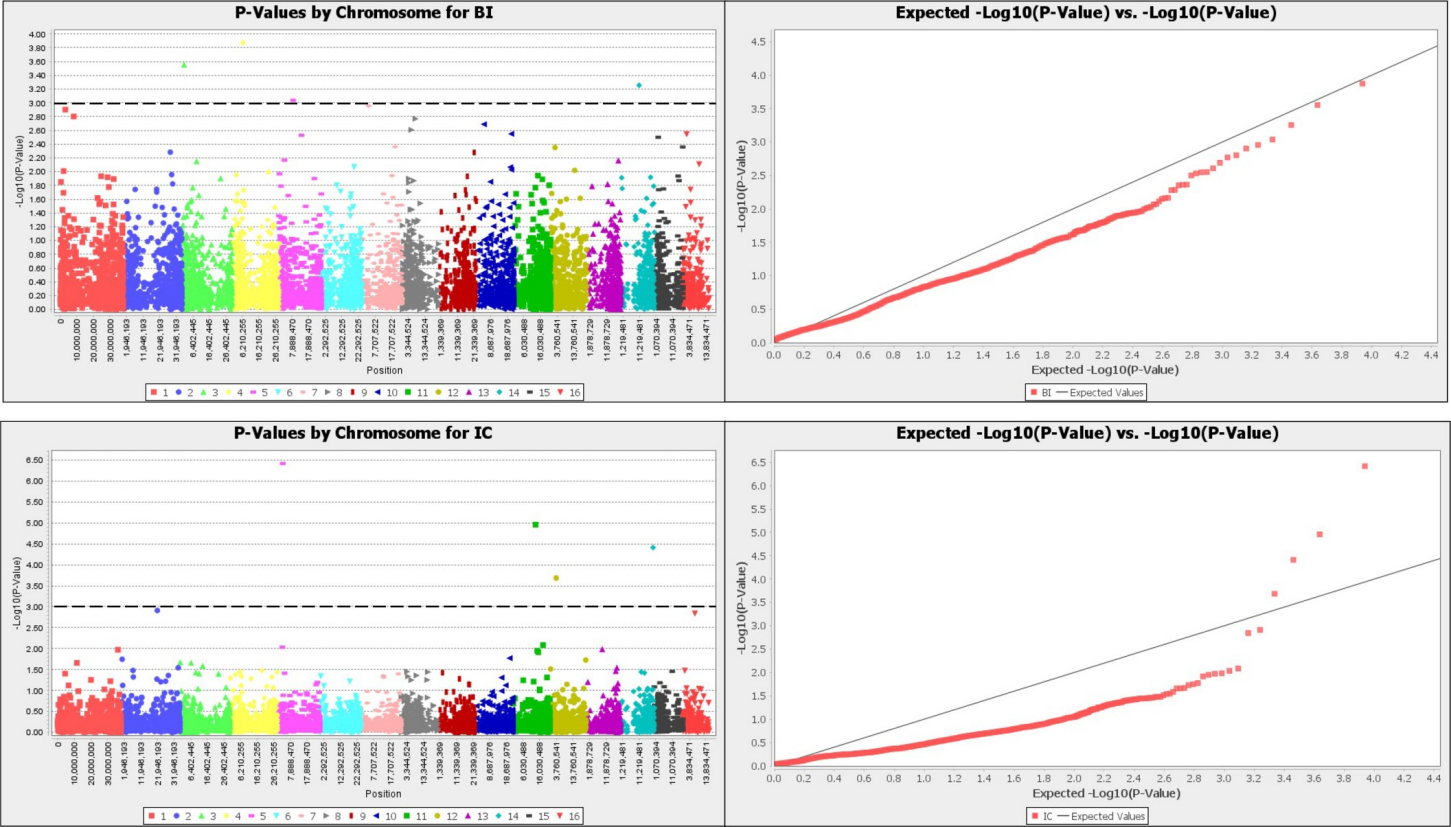
Manhattan plot and QQ plot for branching index (BI), inflorescence color (IC), leaf pigmentation (LP) and leaf shape (LS) of 16 amaranth species.

**Fig 6:**
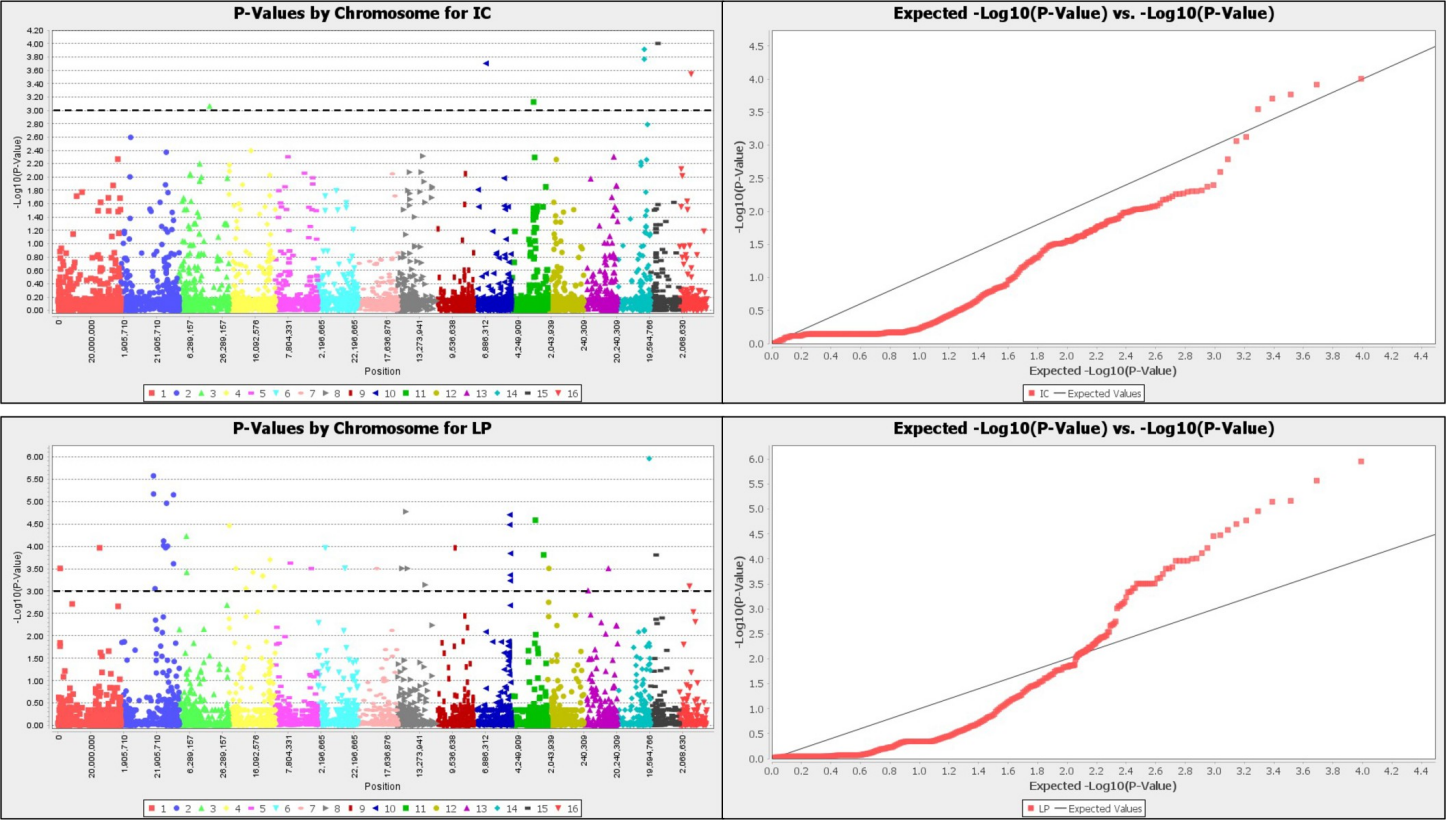
Manhattan plot and QQ plot for inflorescence color (IC), leaf pigmentation (LP), petiole pigmentation (PP) and stem pigmentation (SP) of 118 *A*. *tricolor* accessions.

Furthermore, the mapping of this amaranth panel with the reference genome, *A*. *hypochondriacus* [14] identified twelve putative candidate genes with functional protein. These markers had low phenotypic variation (<20%) evaluated on all respective traits. The Manhattan plots of–log(p)>3 and the Q-Q plots of these traits are presented in Figs 5 and 6.

## Discussion

The evaluation of molecular markers and morphological traits was carried out on single plants to retain homogeneity of germplasm, as morphological variations were observed among amaranth plants within one collection. The evaluation of single plants is necessary as amaranth has high phenotypic plasticity which appears to be heterogamous in field plantings and thus adapts easily to the environmental changes, even though selection within cultivar/landrace has the possibility to be infertile [34]. The capacity of amaranth to have wide genetic variability provides new prospects in the development of new crop varieties. Therefore, the construction of population structure in amaranth through a combination of morphological and molecular data is needed in order to develop a framework for future breeding programmes.

GBS data can have a high proportion of missing values [16] and the number of SNPs retained for the analysis depends on the quality control method [35]. In this study, a large number of SNP markers (74,306 SNP) were generated through the DArTSeq method, a non-reference based approach (*de novo*) using the *PstI* and *MseI* endonucleases in the library preparation step. After aligning the sequence tags against the very high quality and full length macromolecules of the *A*. *hypochondriacus* reference genome for SNP locations [14], the DArTSeq was able to generate relatively large numbers of SNP marker which could be mapped to the *A*. *hypochondriacus* genome (63,821 SNP) and suggests that DArTSeq as a technique should provide for full genome coverage. The number of SNP loci discovered in this study compared favorably with previous GBS studies generated in amaranth species that used *ApeKI* single enzyme cutting combined with deep reference-based assembly methodsm [17] as well as studies that used two library preparations via reference-based and non-reference based assembly methods [15, 16]. After filtration, the range of polymorphic SNP markers used in this study was comparable with other findings, such as 3,974 DArTSeq SNPs successfully used for population structure of 67 wild Galapagos tomato accessions (*Solanum cheesmaniae* and *S*. *galapagense*) [36] and 3,956 DArTSeq SNPs used in 80 macadamia accessions (*Macadamia integrifolia*, *M*. *tetraphylla* and hybrids) [37].

Population structure analysis on 16 amaranth species generates consistent taxonomic classification of amaranth sub-genera which was previously defined using seeds, inflorescence and floral characteristics [7, 38]. Three amaranth sub-genera *Amaranthus Amaranthus*, *Amaranthus Acnida* and *Amaranthus Albersia* were well defined in this study, consistent with other GBS findings by [15]. Subgenus *Amaranthus*, comprised of grain amaranth (*A*. *hypochondriacus* and *A*. *cruentus*) and its weed progenitor (*A*. *hybridus*) were distinguished in Pop 3. Subgenus *Albersia*, which comprised of vegetable amaranth including *A*. *tricolor* were distinguished in Pop 1 and Pop 2, together with six out of seven *A*. *blitum* accessions, three *A*. *graecizans* accessions and four of six *A*. *viridis* accessions. Meanwhile, species belonging to subgenus *Acnida*, which comprised of weedy amaranth, *A*. *spinosus* and *A*. *palmeri* were diverse between the three sub-populations. Another important finding was *A*. *hybridus* that belonged to sub-genus *Amaranthus* was split into sub-genus *Albersia*. *A*. *hybridus* is the direct ancestor of cultivated grain amaranth species [39, 40], and the split of accessions identity could be due to inter-varietal hybridization. Weedy amaranth, *A*. *spinosus* is a cross-pollinated and subsequent gene flow between populations may occur more rapidly than the primarily self-pollinated amaranth species [40]. Lee et al. [47] also have stated that varying amounts of outcrossing and frequent interspecific and inter-varietal hybridization have occurred in amaranth accessions even though it is self-pollinated. Therefore, this could explain the admixture between amaranth species. Besides, this study found that weedy amaranth possessed more unique SNPs per accession than grain amaranth evidently perhaps suggesting that weedy species have had far less selection pressure than the cultivated grain species, which is useful from a breeding perspective

There is also genetic differentiation between grain and vegetable amaranth in this study, which has also been observed in many molecular markers studies, including AFLP [41], SSR [23, 42, 43] and GBS [15], although those studies incorporated far fewer *A*. *tricolor* accessions. This genetic analysis has not only revealed duplicates and genetically closely related individuals, but also allowed categorization of accessions into the correct species. In this study, two *A*. *tricolor* accessions (AV-TRI 20 and AV-TRI 28) from Asia deviated from the *A*. *tricolor* clade and were grouped together with sub-genera *Amaranthus*, which mainly belonged to grain and weed amaranths. There are two assumptions for this finding, either the two amaranths were incorrectly identified as *A*. *tricolor* [17] or were originally a landrace that was grown in a region where grain amaranth was traditionally cultivated over a long time through seeds exchange [44–46]. In a previous study, GBS accurately identified *A*. *caudatus* accession PI 490752, characterized as *A*. *hypochondriacus* by 11 SSR markers [39], but it should be assigned into the *A*. *caudatus* group [17]. Therefore, re-analysis should be carried out for these two *A*. *tricolor* accessions, with addition of larger morphological dataset, which could correct the possible misclassification. The occurrence of admixed/hybrid genotypes may indicate frequent hybridization or introgression events. An experiment based on SSR markers by [23] revealed that *A*. *tricolor* accessions did not correlate between groups which may imply that *A*. *tricolor* had larger genetic variation. There was also uncertainty in positioning phylogeny of *A*. *tricolor* accessions among amaranth species, although *A*. *tricolor* accessions were grouped together in a clade [15]. *A*. *tricolor* had by far the largest estimated genome size (782.7Mbp) among 35 amaranth species, and this suggests that polyploidization likely influenced the genome size of this species [15].

In this study, the species groupings were independent of the accession’s geographical origin, contradicting previous GBS findings [15–17]. In previous studies, geographical patterns demonstrate that comprehensive origin sampling can assist in understanding the evolution of the species as shown by a strong split of geographic pattern in *A*. *hybridus* between accessions from Central and South America, which later supports the hypothesis that two different lineages were the ancestors of the grain amaranth [15]. In this study, the genetic differentiation between species and geographical origin was weak, although a strong split of geographical pattern was observed in *A*. *hybridus* where accessions from America and Africa were divided into two clusters, which may explain the genetic differentiation of *hybridus* complex [23]. This is probably due to the cosmopolitan nature of the genus, or the results of human activities such as breeding and resource exchange [47]. While the current study used a different restriction endonuclease frequent cutter for construction of the genomic representations sequenced, the biased number of accession per species could contribute to the lack discrimination of geographical origin and species level. This was also observed in 3,431 DArTSeq SNPs used to conduct genetic diversity in 89 safflower accessions (*Carthamus tinctorius* L.), in which the SNPs showed weak correlation between safflower diversity pattern and origins, when compared with to a larger SNP dataset [48]. However, for a large set of 118 *A*. *tricolor* accessions, genetic differentiation of Bangladeshi accessions was clear as they clustered together and had distinct morphological characters.

The closely related *A*. *hypochondriacus* genome was used as the genome reference for association mapping as no *A*. *tricolor* genome is available to date. The utilities of the reference quality genome were demonstrated in two ways, i.e. chromosomal evolution and mapping of genetic locus responsible for stem color, hence ample support to clarify the scientific understanding of a useful agricultural trait in amaranth. The highly significant MTA found in morphological traits in this study illustrate how this DArTSeq data can provide high resolution genome coverage for mapping opportunities. However, the most significant associations detected in the MLM model had a lower threshold (−log(p−value)<4, although the mixed model was superior, it still could be lead to at least one false negative and false positive [49]. This could be due to the use of different amaranth species (*A*. *hypochondriacus*) as a reference genome instead of the *A*. *tricolor* genome. The difficulty of working with plant genomes is that they are highly repetitive and feature extensive structural variation between members of the same species, mostly attributed to their active transposons [50] and chromosomal rearrangements. For example, in the well-studied species *Arabidopsis thaliana*, natural accessions are missing 15% of the reference genome, indicating a similar fraction would be absent from the reference, but present in other accessions [51]. Moreover, although *A*. *thaliana* has a small (140 Mb) and not very repetitive genome compared to many other plants, SNPs may be assigned to incorrect positions due to sequence similarity shared between unlinked loci [52]. Therefore, more extensive structural variation would be expected in a larger *A*. *tricolor* genome, which contain a higher proportion of repeats and has undergone ancient and recent rounds of polyploidization [15].

### Conclusions

The findings in this study demonstrated that the DArTSeq SNP data generated from 181 amaranth accessions comprised of 16 species was capable of differentiating vegetable amaranth, *A*. *tricolor* from grain and wild amaranth species. The species groupings were independent of accessions’ geographical origin. This is likely a result of germplasm origin being registered as where the seeds were donated from, which may not be the actual origin of the accession or movement of germplasm in recent historical time. For a larger *A*. *tricolor* data set, there was likelihood that a good differentiation of *A*. *tricolor* could be achieved based on a combined analysis of molecular markers, geographical origin and morphological traits. GWAS used to conduct a pilot genome association for 10 morphological traits demonstrates the potential effectiveness of the amaranth diversity panel for trait dissection. The high degree of morphological variation observed in amaranth may be beneficial in terms of its adaptive capabilities in different climatic conditions.

## Supporting information

S1 Table DArTSeq SNP reads from the 188 amaranth accessions for18 species. (XLSX)

S2 Table 10 morphological traits of *A*. *tricolor* subset observed under shade-house conditions. (XLSX)

S1 Fig Cross-entropy plot for (a) first population structure: 181 amaranth accessions of 16 species and (b) second population structure: 118 *A*. *tricolor* accessions. A range of K = 1:8 was tested and K = 3 was chosen as the cross-entropy curve exhibits a plateau in both datasets.
(TIF)
