# Supplementary figures and images for: Genetic diversity analysis and marker-trait associations in Amaranthus species
Source: PLoS One. 2022 May 12;17(5):e0267752. doi: 10.1371/journal.pone.0267752 (PMC9098028; doi:10.1371/journal.pone.0267752)

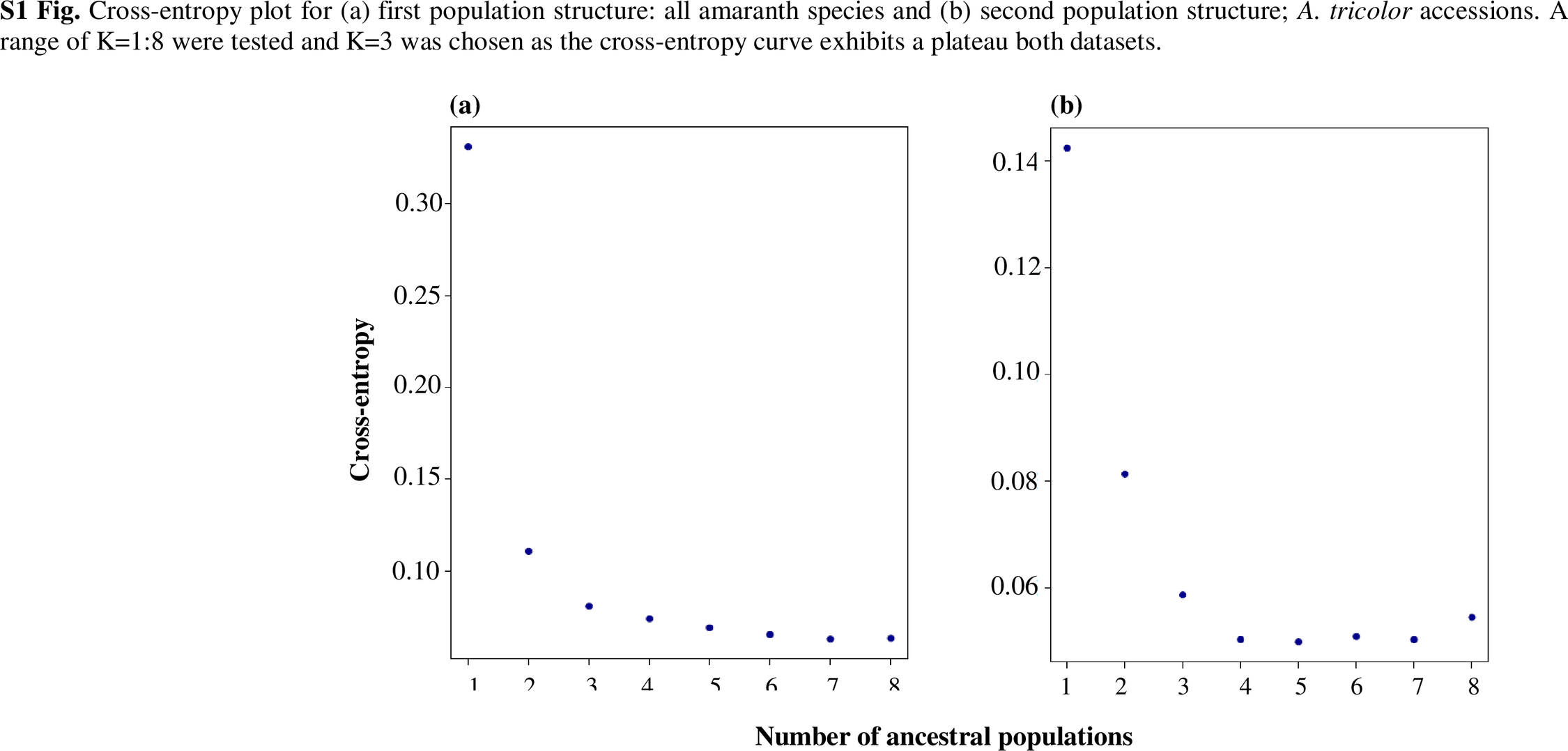

Supplement: S1 Fig — Cross-entropy plot for (a) first population structure: 181 amaranth accessions of 16 species and (b) second population structure: 118 A. tricolor accessions. A range of K = 1:8 was tested and K = 3 was chosen as the cross-entropy curve exhibits a plateau in both datasets. (TIF) [file pone.0267752.s003.tif]
